# The structures of *Arabidopsis* Deg5 and Deg8 reveal new insights into HtrA proteases

**DOI:** 10.1107/S0907444913002023

**Published:** 2013-04-11

**Authors:** Wei Sun, Feng Gao, Haitian Fan, Xiaoyue Shan, Renhua Sun, Lin Liu, Weimin Gong

**Affiliations:** a Laboratory of Non-coding RNA, Institute of Biophysics, Chinese Academy of Sciences, 5 Datun Road, Chaoyang District, Beijing 100101, People’s Republic of China; b Photosynthesis Research Center, Key Laboratory of Photobiology, Institute of Botany, Chinese Academy of Sciences, 20 Nanxincun, Haidian District, Beijing 100093, People’s Republic of China; c University of Chinese Academy of Sciences, 19A Yuquan Road, Beijing 100049, People’s Republic of China

**Keywords:** protease–chaperones, protein quality control, PDZ domains, oligomerization

## Abstract

Plant Deg5 and Deg8 are two members of the HtrA proteases, a family of oligomeric serine endopeptidases that are involved in a variety of protein quality-control processes. These two HtrA proteases are located in the thylakoid lumen and participate in high-light stress responses by collaborating with other chloroplast proteins. Deg5 and Deg8 degrade photodamaged D1 protein of the photosystem II reaction centre, allowing its *in situ* replacement. Here, the crystal structures of *Arabidopsis thaliana* Deg5 (S266A) and Deg8 (S292A) are reported at 2.6 and 2.0 Å resolution, respectively. The Deg5 trimer contains two calcium ions in a central channel, suggesting a link between photodamage control and calcium ions in chloroplasts. Previous structures of HtrA proteases have indicated that their regulation usually requires C-terminal PDZ domain(s). Deg5 is unique in that it contains no PDZ domain and the trimeric structure of Deg5 (S266A) reveals a novel catalytic triad conformation. A similar triad conformation is observed in the hexameric structure of the single PDZ-domain-containing Deg8 (S292A). These findings suggest a novel activation mechanism for plant HtrA proteases and provide structural clues to their function in light-stress response.

## Abstract 2

The crystal structures of *Arabidopsis* Deg5 and Deg8 have been determined to resolutions of 2.6 and 2.0 Å, respectively, revealing novel structural features of HtrA proteases.

## 1. Introduction

HtrA (high-temperature requirement A) proteases are a family of ATP-independent trypsin-like serine endopeptidases that belong to MEROPS subfamily S1C and are involved in protein quality control (PQC) in both prokaryotes and eukaryotes (Rawlings *et al.*, 2008 ▶; Clausen *et al.*, 2002 ▶; Ehrmann & Clausen, 2004 ▶). They have an N-terminal protease domain and 0–3 PDZ (PSD95/Dlg1/ZO-1) domain(s) at the C-­terminal end. The functional unit of the HtrA proteases is a trimer, with three protease domains forming a central core. The physiological importance of the HtrA proteases can be inferred from their wide distribution from bacteria to humans. Three HtrA proteases from *Escherichia coli*, DegS, DegP and DegQ, are the best-characterized prokaryotic members (Singh *et al.*, 2011 ▶). They are located in the periplasmic space and are involved in essential PQC processes. Human HtrA1 and HtrA2 are the most thoroughly studied eukaryotic members and both have been discovered to play key roles in development and disease (Clausen *et al.*, 2011 ▶).

Their lack of motility renders plants constantly susceptible to environmental stresses. To deal with photodamage to their photosynthetic machinery, plants have evolved a sophisticated PQC system (Sakamoto, 2006 ▶; Eberhard *et al.*, 2008 ▶). The members of the HtrA proteases have been identified as critical components of the chloroplast PQC system (Schuhmann *et al.*, 2012 ▶; Komenda *et al.*, 2012 ▶). Among the 16 *Arabidopsis* HtrA proteases that have been annotated, Deg1, Deg5 and Deg8 are in the chloroplast thylakoid lumen (Schuhmann & Adamska, 2012 ▶). Deg1 has been reported to be involved in photoinhibition repair and photosystem II (PSII) assembly through its dual protease and chaperone activity (Kapri-Pardes *et al.*, 2007 ▶; Sun, Ouyang *et al.*, 2010 ▶). Deg5 and Deg8 are required for efficient PSII repair under light stress and recombinant Deg8 is proteolytically active with β-casein as the substrate, whereas Deg5 demonstrates no β-casein degradation activity (Sun *et al.*, 2007 ▶; Kato *et al.*, 2012 ▶). Deg1, Deg5 and Deg8 together with two stromal HtrA members, Deg2 and Deg7 (Haussühl *et al.*, 2001 ▶; Sun, Fu *et al.*, 2010 ▶), participate in degradation of damaged reaction-centre protein D1 in the PSII complex during light stress.

The structures of members of the HtrA proteases from *E. coli* (DegS, DegP and DegQ) and of human HtrA1 and HtrA2 have provided detailed insights into their activation mechanisms. Trimeric DegS requires the binding of stress-signalling peptides to its PDZ domain for allosteric activation (Wilken *et al.*, 2004 ▶; Sohn *et al.*, 2007 ▶), while DegP and DegQ form large oligomers with encapsulated substrate (Krojer *et al.*, 2008 ▶; Kim *et al.*, 2011 ▶; Malet *et al.*, 2012 ▶). Unlike DegS, the HtrA1 trimer and the HtrA2 trimer can degrade substrates directly without any other cofactors (Truebestein *et al.*, 2011 ▶; Li *et al.*, 2002 ▶).

Recent structural studies on *Arabidopsis* Deg1 have suggested a pH-dependent activation pathway (Kley *et al.*, 2011 ▶). It has been shown that loops LA, L1, L2, L3 and LD play direct roles in the regulation of Deg1 activity. At acidic pH, Deg1 trimers dimerize through interactions between the PDZ domain and loop LA of the protease domain, leading to a fixed position of the PDZ domain and establishing a PDZ–L3–LD interaction network. As it is naturally devoid of a PDZ domain, Deg5 should employ a different activation mechanism. Although Deg8 is the closest homologue of Deg1 in the *Arabidopsis* genome (Schuhmann & Adamska, 2012 ▶), these two enzymes are functionally different. Deg1 is constitutively expressed and is required for plant growth, while Deg8 is sensitive to high light levels and together with Deg5 does not contribute significantly to the degradation of PSII D1 under low-light conditions (Kato *et al.*, 2012 ▶). In addition, Deg5 and Deg8 have a synergistic effect on PSII D1 degradation during high light stress (Sun *et al.*, 2007 ▶). All of these facts indicate that Deg5 and Deg8 utilize different mechanisms to regulate protease activity and the structural basis behind them needs to be examined.

Here, we report the crystal structures of Deg5 (S266A) and Deg8 (S292A) at 2.6 and 2.0 Å resolution, respectively. Both structures represent proteolytically incompetent states. The catalytic triad in both structures is malformed by rotation of the conserved histidine. We also find that the Deg5 trimer can bind two calcium ions. These findings provide novel structural evidence of the differing activities and regulation mechanisms of HtrA proteases.

## 2. Materials and methods

### 2.1. Expression, purification and crystallization

The expression, purification, crystallization and preliminary diffraction analysis of Deg5 (S266A) and Deg8 (S292A) have been described previously (Fan *et al.*, 2012 ▶; Shan *et al.*, 2013 ▶).

### 2.2. Data collection and structure determination

All data collections were performed on beamline BL17U at Shanghai Synchrotron Radiation Facility (SSRF) using a MAR 225 CCD detector (MAR Research). Data were processed with *HKL*-2000 (Otwinowski & Minor, 1997 ▶).

Deg5 crystallized in space group *C*2, with three molecules per asymmetric unit that are related by a noncrystallographic symmetry threefold axis. The structure of Deg5 (S266A) was solved by the molecular-replacement method using *MOLREP* (Vagin & Teplyakov, 2010 ▶) within the *CCP*4 suite (Winn *et al.*, 2011 ▶). HtrA from *Thermotoga maritima* (PDB entry 1l1j; Kim *et al.*, 2003 ▶) was used as the search model. The initial model was built with *ARP*/*wARP* (Cohen *et al.*, 2008 ▶) and amino acids were then added manually in *Coot* (Emsley *et al.*, 2010 ▶). Refinement was performed with *REFMAC*5 (Murshudov *et al.*, 2011 ▶). Noncrystallography symmetry restraints were used. The final *R* and *R*_free_ were 18.2% and 23.1%, respectively.

Deg8 (S292A) crystals were soaked in cryoprotectant (12% 2-methyl-2,4-pentanediol) and data collection was performed at a wavelength of 0.9792 Å in a nitrogen stream at 100 K. The Deg8 crystal belonged to space group *C*2 and one asymmetric unit contained three Deg8 molecules, which are also related by a noncrystallographic symmetry threefold axis. The Deg8 (S292A) structure was also solved by molecular replacement and the search model for Deg8 (S292A) was the structure of *Arabidopsis* Deg1 (PDB entry 3qo6; Kley *et al.*, 2011 ▶), including both the protease and PDZ domains. The model was refined with *PHENIX* (Adams *et al.*, 2010 ▶) and *CNS* (Brünger *et al.*, 1998 ▶) and was built using *Coot* (Emsley *et al.*, 2010 ▶) based on the *F*_o_ − *F*_c_ electron-density map. Further refinement was performed with *REFMAC*5 (Murshudov *et al.*, 2011 ▶). The final *R* and *R*_free_ were 19.8% and 23.5%, respectively.

The final models of Deg5 (S266A) and Deg8 (S292A) were validated with *PROCHECK* (Laskowski *et al.*, 1993 ▶). Statistics of data collection and refinement are summarized in Table 1 ▶.

### 2.3. Inductively coupled plasma mass spectrometry (ICP-MS)

Analysis of calcium content was carried out using ICP-MS (Thermo Scientific) at the Tsinghua University Analysis Center (Beijing, People’s Republic of China). Purified Deg5 (S266A) was dialyzed thoroughly against buffer consisting of 20 m*M* Tris–HCl pH 8.0, 150 m*M* NaCl before ICP-MS. To obtain calcium-stripped samples, 5 m*M* EDTA was added to the dialysis buffer. The ICP-MS data are summarized in Table 2 ▶.

### 2.4. Oligomeric state analysis

10 µ*M* protein was buffered in sodium phosphate pH 6.0 or 8.0, 150 m*M* NaCl and was incubated on ice for at least 1 h. Samples were then loaded onto a Superdex 200 10/300 GL (GE Healthcare) size-exclusion column and their molecular weights were calculated using a calibration curve determined using calibration standards (GE Healthcare).

### 2.5. Proteolytic activity assay

For each assay, 100 µg β-casein was incubated in 200 µl 50 m*M* sodium phosphate pH 6.0, 50 m*M* NaCl with or without 20 µg recombinant Deg8 or 2 µg recombinant Deg1 at 310 K. The reaction mixtures at different time points were subjected to SDS–PAGE on a 12% acrylamide gel.

## 3. Results and discussion

### 3.1. Structure of *A. thaliana* Deg5 (S266A)

**Figure 1:**
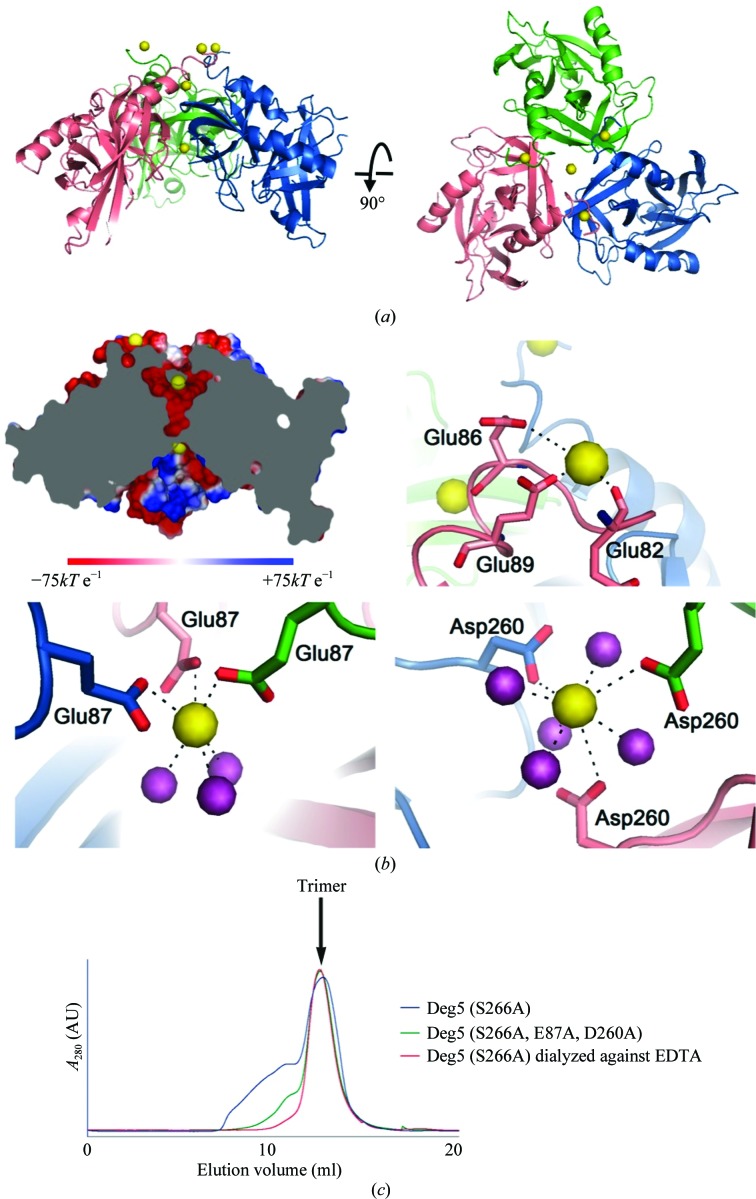
Overall structure of Deg5 (S266A) and binding of Ca^2+^ ions. (*a*) Side and top views of the Deg5 (S266A) trimer with each protomer coloured differently. (*b*) Ca^2+^ ions binding to Deg5 (S266A). Top and left, electrostatic potential distributions of the inner surface of the trimer. Electrostatic potential was calculated using *PyMOL*. Blue and red represent positive and negative charge potentials on a scale between +75 and −75*kT* e^−1^, respectively. Top and right, Ca^2+^ ions binding to the convex side of the trimer. Bottom, Ca^2+^ ions binding to the central channel of the Deg5 trimer. Ca^2+^ ions and waters are shown as spheres and are coloured yellow and purple, respectively. Residues interacting with Ca^2+^ ions are shown as sticks and are labelled. Salt bridges formed between Deg5 and Ca^2+^ ions are presented as black dashed lines. (*c*) Analytical SEC of Deg5 (S266A) (blue), Deg5 (S266A) dialyzed against EDTA (red) and Deg5 (S266A/E87A/D260A) triple mutant (green). The trimeric peak is indicated by an arrow.

His147, Asp188 and Ser266 constitute the catalytic triad of Deg5. To avoid self-degradation of wild-type Deg5 during crystallization, Ser266 was mutated to alanine (Fan *et al.*, 2012 ▶). The crystal of Deg5 (S266A) belonged to space group *C*2 and contains a trimer with a threefold noncrystallographic axis of symmetry in the asymmetric unit (Fig. 1 ▶ *a*). The trimer has a shallow funnel shape and the catalytic triad is located on the concave side of the funnel. Five Ca^2+^ ions are observed per trimer. Three are on the convex side and each interacts with the N-terminal acidic residues of each protomer. The other two are located on the noncrystallographic threefold axis, along which a channel is formed (Figs. 1 ▶ *a* and 1 ▶ *b*). In the crystal two Deg5 (S266A) trimers associate with each other through their convex surfaces. In the back-to-back hexamer the interactions are mainly hydrogen bonds and salt bridges involving the Ca^2+^ ions on the surfaces. The molecular weight of Deg5 (S266A) as measured by size-exclusion chromatography (SEC) and dynamic light scattering is approximately 100 kDa, indicating that the trimer is the solution species and that two trimers only associate in the crystal.

Trimerization of the protomers is mediated through hydrophobic interactions, hydrogen bonds and a π–cation interaction. A hydrophobic interface is formed by residues of two regions. One includes Leu231 and Ile233 on β9, Phe95 on the N-terminal helix α1, Val235* on β9 (where the asterisk denotes the participation of the neighbouring protomer) and Val214* on the loop before β8; the other includes Tyr225 on loop LD and Val296* and Phe298* on β13 (Supplementary Fig. S1*a* 1). Hydrogen-bond interactions are found in the area around the hydrophobic interface and include the following pairs: Glu83–Tyr274*, Glu85–Arg90*, Glu88–Ser217*, Glu88–Ser273*, Asn91–Gly215*, Gln96–Arg213*, Leu231–Ser237* and Ile233–Asp258* (Supplementary Fig. S1*b*). Moreover, Tyr227 donates π-­electrons to the H atom on the N^∊^ atom of Arg241*, which also contributes to trimer stabilization (Supplementary Fig. S1*c*).

### 3.2. Ca^2+^ ions in the central channel of the Deg5 trimer

The electrostatic surface of the central channel in the Deg5 (S266A) trimer is highly acidic (Fig. 1 ▶ *b*, top and left). The carboxyl groups of Glu87 and Asp260 stabilize two Ca^2+^ ions at both ends of the channel (Fig. 1 ▶ *b*, bottom). ICP-MS analysis of the Deg5 (S266A) solution sample quantified that the Ca^2+^:protomer ratio was 0.60, corresponding to 1.8 Ca^2+^ ions per trimer (Table 2 ▶). In the crystal there are five Ca^2+^ ions. The three excess Ca^2+^ ions bound to the N-terminal acidic residues of Deg5 (S266A) (Fig. 1 ▶ *b*, top and right) were probably introduced during crystallization. To test whether the central two Ca^2+^ ions coordinated by acidic residues (Glu87 and Asp260) are required for Deg5 trimerization, we mutated both Glu87 and Asp260 to alanine. SEC revealed that the mutant (E87A/D260A/S266A) exists predominantly as a trimer (Fig. 1 ▶ *c*). ICP-MS analysis of this triple mutant quantified that the Ca^2+^:protomer ratio was 0.12, corresponding to 0.36 Ca^2+^ ions per trimer. These results suggested that the two Ca^2+^ ions observed in the crystal structure are also accommodated by the two acidic residues in solution.

Deg5 may act as Ca^2+^-storage protein in chloroplasts. To test this possibility, Deg5 (S266A) was dialyzed against 5 m*M* EDTA and the Ca^2+^:protomer ratio was analyzed by ICP-MS (Table 2 ▶). The ratio decreased to 0.037 Ca^2+^ ions per protomer and analysis of the oligomeric state using SEC confirmed that Deg5 remained as a trimer (Fig. 1 ▶ *c*). These results indicated that the Ca^2+^ ions can be readily stripped by EDTA and are dispensable for Deg5 trimerization, indicating a potential role of Deg5 in calcium signalling. Calcium signals are critical for plant development and stress adaptation (Kudla *et al.*, 2010 ▶). A recent publication proposed that the thylakoid luminal calcium concentration is a signalling mechanism for PSII oxygen-evolving complex assembly and D1 repair (Lohmiller *et al.*, 2012 ▶). The Deg5 (S266A) structure reported here thus provides new clues to the function of Deg5 in PSII quality control. Given the importance of chloroplasts in calcium signalling (Stael *et al.*, 2012 ▶), the biological function of Ca^2+^ ions in the Deg5 central channel awaits further studies.

### 3.3. Deg5 active site

**Figure 2:**
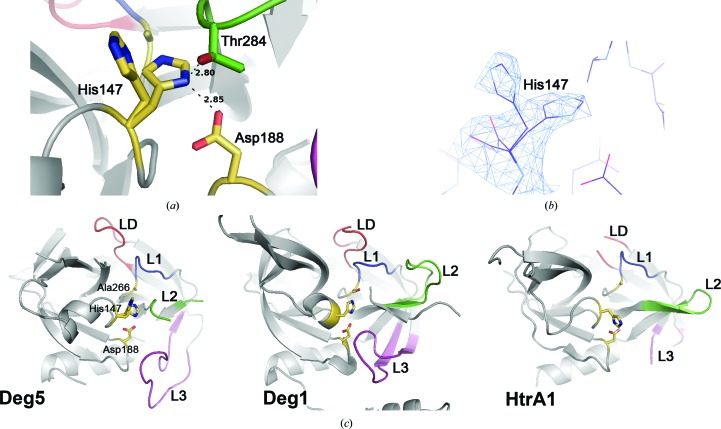
Structural comparison of the active sites of Deg5 (S266A), Deg1 and HtrA1. (*a*) Catalytic triad of Deg5 (S266A) (yellow). Hydrogen bonds between His147 and Asp188 and between His147 and Thr284 are presented as black dashed lines. The catalytic triad and Thr284 from loop L2 are shown in stick mode. (*b*) Alternative conformation of His147. His147 is displayed together with electron density calculated using 2*F*_o_ − *F*_c_ coefficients and contoured at 0.7σ as a blue mesh. (*c*) Structural comparison of catalytic triads and activation loops of Deg5 (S266A), Deg1 (PDB entry 3qo6) and HtrA1 (PDB entry 3num; Truebestein *et al.*, 2011 ▶). Catalytic triads are shown in stick mode and are coloured yellow. Loops LD, L1, L2 and L3 are coloured red, blue, green and magenta, respectively.

In the Deg5 (S266A) trimer, the side chain of the catalytic His147 has two conformations (Figs. 2 ▶ *a* and 2 ▶ *b*). One contains the conserved hydrogen bond between His147 and Asp188, while the alternative conformation differs from that in any reported HtrA structure (Clausen *et al.*, 2011 ▶). The χ_1_ angle of His147 in the alternative conformation is rotated anti­clockwise by ∼120°, preventing formation of the His147–Asp188 hydrogen bond, which is an unusual conformation compared with other distorted HtrA catalytic triads (Krojer *et al.*, 2002 ▶; Kim *et al.*, 2003 ▶; Wrase *et al.*, 2011 ▶). It resembles the catalytic histidine of human HtrA1 (Truebestein *et al.*, 2011 ▶; Eigenbrot *et al.*, 2012 ▶), but in that case the imidazole ring is rotated clockwise around the χ_1_ axis (Fig. 2 ▶ *c*).

In Deg5 (S266A), residues 111–125 from loop LA, 155–159 from loop LB and 287–295 from loop L2 cannot be traced in the electron-density map. These loops, together with loop L3 (241–251), which has significantly high *B*-factor values, are flexible in the crystal. Loops L1, L2 and LD are defined as the activation domain, the proper conformation of which indicates activated HtrA proteases (Krojer *et al.*, 2002 ▶, 2010 ▶; Kley *et al.*, 2011 ▶), with L1 being linked to oxyanion-hole formation, L2 to the substrate-specificity pocket and LD being defined as the activation loop. Thr284 of loop L2 stretches close to the active site and forms a hydrogen bond to His147 (Fig. 2 ▶ *a*) which blocks substrate from approaching the catalytic triad. The missing electron density for loops including L2, together with the distorted His147, indicates that the Deg5 structure shown here is of an inactive form. Deg5 may become proteolytically active after the conformation of loops L1 and L2 is changed by allosteric or substrate activation.

### 3.4. Structure of *A. thaliana* Deg8 (S292A)

**Figure 3:**
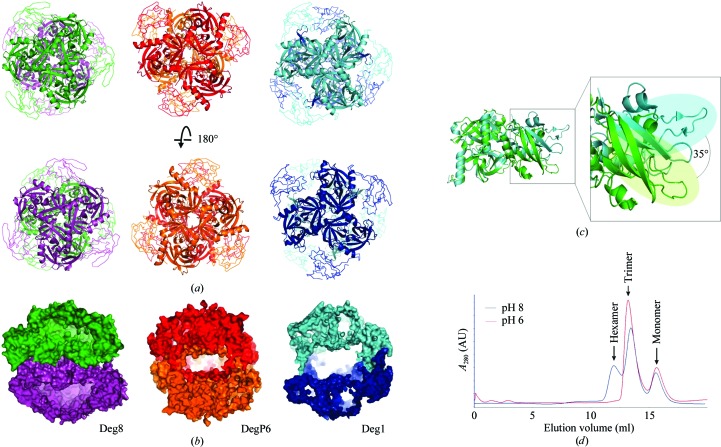
Structure of Deg8 (S292A) in comparison with DegP6 and Deg1 and oligomeric state analysis. (*a*) Structures of Deg8 (S292A) (green and magenta), DegP6 (PDB entry 1ky9; red and orange; the second PDZ domain is not shown for clarity; Krojer *et al.*, 2008 ▶) and Deg1 (PDB entry 3qo6; cyan and blue). Protease domains and PDZ domains are shown in cartoon and ribbon modes, respectively. (*b*) The cavity formed in Deg8, DegP6 (PDB entry 1ky9; molecule *B*) and Deg1 (PDB entry 3qo6). (*c*) Structural comparison of protomers of Deg8 (S292A) (green) and Deg1 (PDB entry 3qo6; cyan); the difference in PDZ-domain orientations is indicated. (*d*) Analytical SEC of Deg8 at pH 6.0 (red line) and pH 8.0 (blue line). Different oligomeric states are labelled above the peaks.

The crystallized Deg8 (S292A) contains 358 amino-acid residues (Leu91–Ser448), of which residues 104–448 could be defined in the electron-density map. Deg8 (S292A) forms a hexamer in the crystals (Figs. 3 ▶ *a* and 3 ▶ *b*). Interestingly, when superimposing one trimer on Deg1 or DegP6 (DegP hexamer), the orientation of the opposite trimer of the Deg8 protease domains differs from that of Deg1 but is more similar to that of DegP6 (Fig. 3 ▶ *a*). Despite the higher similarity between Deg1 and Deg8, when compared with Deg1 (Kley *et al.*, 2011 ▶) the orientation of the Deg8 PDZ domain changes by ∼35° along the major inertia axis (Fig. 3 ▶ *c*). As such, the Deg8 PDZ domain is flattened out from the trimeric core made by the protease domain and the concavity of the trimeric funnel is shallower than in Deg1. The internal cavity of Deg8 (S292A) is smaller than that of the inactive DegP hexamer (Krojer *et al.*, 2002 ▶; Fig. 3 ▶ *b*), with their volumes being 46 143 and 69 527 Å^3^, respectively, as calculated by 3*V* (Voss & Gerstein, 2010 ▶). Such a compact hexameric assembly would prevent the substrate from accessing the active site.

Trimerization is primarily mediated by hydrophobic interactions and hydrogen bonds. The residues involved in hydrophobic interactions are Val240, Val261, Phe123*, Leu257*, Pro355, Phe251* and Phe253* (Supplementary Fig. S2*a*). Seven pairs of hydrogen bonds are found between two neighbouring protomers: Val232–Phe107*, Ser299–Glu116*, Gln243–Glu116*, Asp284–Val259*, Ser263–Leu257*, Ala318–Asn288* and Arg267–Asp254* (Supplementary Fig. S2*b*). Six pairs of hydrogen bonds are observed between Arg179 and Phe389* from opposite trimers, stabilizing the hexamer (Supplementary Fig. S2*c*). To test the oligomeric state of Deg8 in solution, we performed an analytical SEC assay (Fig. 3 ▶ *d*). The SEC assay showed that Deg8 exists as trimers and monomers at pH 6.0. However, a small fraction of hexamer appeared at pH 8.0, indicating that Deg8 tends to form higher oligomers at basic pH rather than acidic pH (Fig. 3 ▶ *d*). This behaviour differs from that of Deg1, the hexameric form of which is assembled at low pH (Kley *et al.*, 2011 ▶), and from that of Deg2, the hexameric form of which is pH-independent (Sun *et al.*, 2012 ▶).

### 3.5. Deg8 active site

**Figure 4:**
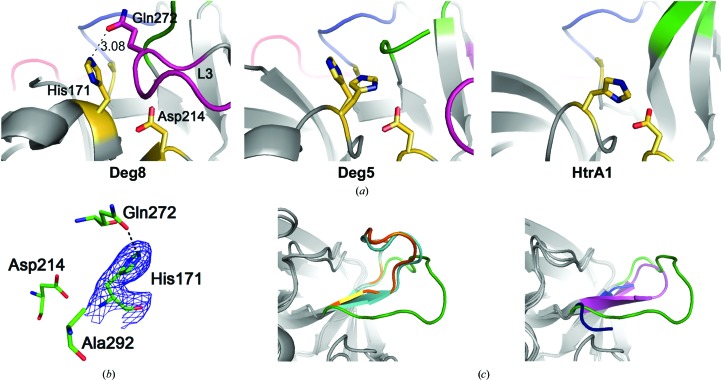
Activation loops and active sites in Deg8 (S292A) and other HtrA proteases. (*a*) Catalytic triad of Deg8 (S292A) (yellow, stick mode) in comparison with Deg5 (S266A) and HtrA1. The hydrogen bond between His171 and Gln272 from loop L3 is presented as a black dashed line. Activation loops are coloured the same as in Fig. 2 ▶(*c*). (*b*) Catalytic triad of Deg8 (S292A). His171 is displayed together with electron density calculated using 2*F*_o_ − *F*_c_ coefficients and contoured at 1.0σ as a blue mesh. (*c*) Structural comparison of loop L2 in Deg8 (S292A) (green), Deg1 (PDB entry 3qo6; cyan; left), DegP24 (PDB entry 3cso; orange; left), Deg5 (blue; right) and HtrA1 (PDB entry 3num; magenta; right).

The catalytic triad of Deg8 consists of His171, Asp214 and Ser292. In the Deg8 (S292A) structure, however, the triad fails to form catalytically competent hydrogen bonds owing to the anticlockwise rotation of the χ_1_ angle of His171 by ∼120° (Figs. 4 ▶ *a* and 4 ▶ *b*), which is similar to that of Deg5 (S266A) and differs from that of HtrA1 (Truebestein *et al.*, 2011 ▶; Eigenbrot *et al.*, 2012 ▶; Fig. 4 ▶ *a*). His171 can form a hydrogen bond to Gln272 of loop L3, a unique feature that was not previously observed in HtrA proteases (Figs. 4 ▶ *a* and 4 ▶ *b*). Moreover, the orientation of loop L2 is quite different from those of other HtrA proteases such as Deg1 (Kley *et al.*, 2011 ▶), the DegP 24-­mer (Krojer *et al.*, 2008 ▶), Deg5 and HtrA1 (Truebestein *et al.*, 2011 ▶; Eigenbrot *et al.*, 2012 ▶; Fig. 4 ▶ *c*). Furthermore, we performed an activity assay on Deg8 using casein as the substrate. The recombinant Deg8 exhibits a remarkably lower proteolytic activity than that of Deg1 in degrading substrate (Supplementary Fig. S2*d*). This incompetent state suggests that to activate Deg8 a new activation mechanism may be employed for rearrangement of the activation domain.

## 4. Conclusions and outlook

Deg5 is a distinct HtrA protease that is naturally devoid of a PDZ domain. Protease activity has not been detected either *in vivo* (Sun *et al.*, 2007 ▶) or *in vitro*. As revealed by the Deg5 (S266A) structure and ICP-MS analysis, trimeric Deg5 can bind two Ca^2+^ ions, suggesting a role in calcium events in chloroplasts. Deg5 (S266A) shows an inactive state in which a novel conformation of the catalytic His147 was observed and Thr284 on loop L2 interacts with His147 and blocks substrate entry. Deg8 contains one PDZ domain that shows a different orientation compared with that in the Deg1 hexamer. The Deg8 (S292A) hexamer has a narrower cavity and a distorted His171 like His147 in Deg5. Our observation of dramatically different conformations of the catalytic histidine reveals motion of this χ_1_ angle and provides new structural insights into the regulation mechanism of chloroplast HtrA proteases.

## Supplementary Material

PDB reference: Deg5, 4ic5

PDB reference: Deg8, 4ic6

Supplementary material file. DOI: 10.1107/S0907444913002023/dw5040sup1.pdf

## Figures and Tables

**Table 1:** Data-collection and refinement statistics for Deg5 (S266A) and Deg8 (S292A)

	Deg5 (S266A)	Deg8 (S292A)
Data collection		
Space group	*C*2	*C*2
Unit-cell parameters (Å, °)	*a* = 109.1, *b* = 126.0, *c* = 83.3, β = 102.9	*a* = 129.5, *b* = 124.2, *c* = 93.3, β = 132.4
Resolution range (Å)	50–2.6 (2.69–2.60)	50–2.0 (2.07–2.00)
No. of reflections (total/unique)	33072/11024	73478/9929
〈*I*/σ(*I*)〉	10.9 (2.3)	25.2 (4.3)
Completeness (%)	99.2 (97.8)	99.3 (100.0)
Multiplicity (%)	3.0 (2.9)	7.4 (7.7)
Wilson *B* factor (Å^2^)	50.2	37.5
*R*_merge_ (%)	9.4 (46.5)	6.7 (47.8)
Structure refinement		
Resolution (Å)	80–2.6	30–2.0
*R*_work_	0.182	0.198
*R*_free_	0.231	0.235
Ramachandran plot (%)		
Favoured region	93.1	96.4
Allowed region	6.1	3.4
Outlier region	0.8	0.2
R.m.s.d. bonds (Å)	0.007	0.007
R.m.s.d. angles (°)	1.026	1.122
Mean *B* value (Å^2^)	51.7	51.8

**Table 2:** ICP-MS analysis of Deg5 with or without EDTA treatment

Protein sample	Ca^2+^ (ng ml^−1^)	Deg5 (ng ml^−1^)	[Ca^2+^]/[Deg5]
Deg5 (S266A)	1553	2.00	0.60
Deg5 (E87A/D260A/S266A)	316	2.00	0.12
Deg5 (S266A) with EDTA treatment	85	1.76	0.037

## Footnotes

1 Supplementary material has been deposited in the IUCr electronic archive (Reference: DW5040). Services for accessing this material are described at the back of the journal.
