# Supplementary material for: The structures of Arabidopsis Deg5 and Deg8 reveal new insights into HtrA proteases
Source: Acta Crystallogr D Biol Crystallogr. 2013 Apr 11;69(Pt 5):830–7. doi: 10.1107/S0907444913002023 (PMC3640471; doi:10.1107/S0907444913002023)
Supplement: Supplementary file 1 [file d-69-00830-sup1.pdf]

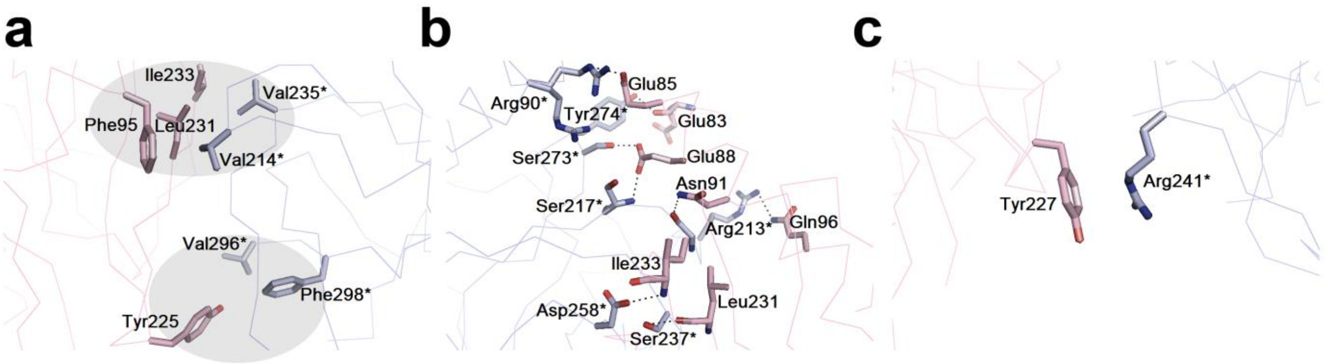

Supplementary figure 1

Hydrophobic interactions (*a*), hydrogen bonds (*b*) and π-electrons (*c*) involved in Deg5 trimer formation. The neighboring Deg5 protomers are shown in light pink and light blue, respectively. Residues involved in trimer formation are shown in stick mode. Two hydrophobic core regions are shadowed gray. Hydrogen bonds are presented as black dash lines.

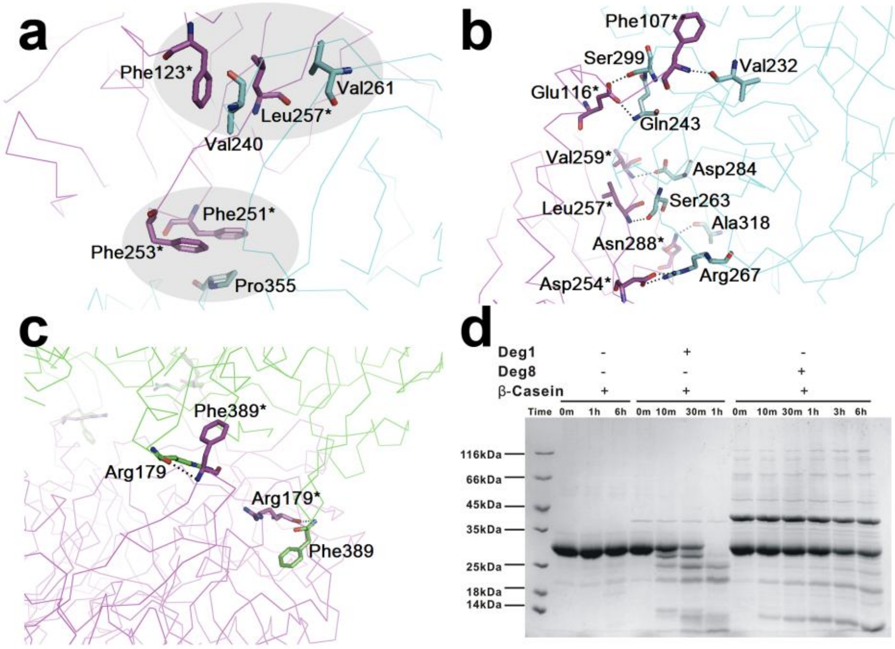

Supplementary figure 2

Interactions involved in Deg8 trimer and hexamer formation and proteolytic activity assays. (*a*)-(*b*) Hydrophobic interactions (*a*) and hydrogen bonds (*b*) involved in Deg8 trimer formation. The neighboring Deg8 protomers are shown in magenta and cyan, respectively. Residues forming hydrophobic interactions or hydrogen bonds are shown in stick mode. Two hydrophobic core regions are shadowed gray. Hydrogen bonds are presented as black dash lines. (*c*) Hydrogen bonds involved in Deg8 hexamer formation. The neighboring Deg8 trimers are shown in green and magenta, respectively. Residues forming hydrogen bonds are shown in stick mode and hydrogen bonds are presented as black dash lines. (*d*) Proteolytic activity assays of Deg1 and Deg8. The reaction mixtures at different time points were subjected to SDS-PAGE (m: min, h: hour).
